# Brucellosis seroprevalence in ovine and caprine flocks in China during 2000–2018: a systematic review and meta-analysis

**DOI:** 10.1186/s12917-018-1715-6

**Published:** 2018-12-12

**Authors:** Xuhua Ran, Xiaohong Chen, Miaomiao Wang, Jiajia Cheng, Hongbo Ni, Xiao-Xuan Zhang, Xiaobo Wen

**Affiliations:** College of Animal Science & Veterinary Medicine, Heilongjiang Bayi Agricultural University, No.5, XinFeng Rd., Saertu District, Daqing City, 163319 Heilongjiang Province China

**Keywords:** Brucellosis, Prevalence, Systematic review, Meta-analysis, China, Ovine and caprine

## Abstract

**Background:**

Brucellosis remains one of the most common zoonotic diseases globally, with more than half million human cases reported annually. Brucellosis is an emerging and re-emerging disease in China since the 1990s. An infectious reservoir constituted by domestic animals with brucellosis, especially ovine and caprine herds, poses a significant threat to public health. The seroprevalence of brucellosis in sheep and goat flocks in a national context is unavailable so far. Therefore, we conducted this systematic review and meta-analysis to assess the overall status of brucellosis in sheep and goats in China in almost two decades.

**Results:**

The pooled prevalence of brucellosis in ovine and caprine flocks in China increased in 2000–2009 (1.00%; 95% CI, 0.70–1.30) to 2010–2018 (3.20%; 95% CI, 2.70–3.60). The seroprevalence of brucellosis in sheep and goat flocks was higher in Eastern China, with 7.00% of positive rate, than that in any other region, especially Shandong province (18.70%). Brucellosis is highly endemic in some local regions. The high prevalence of brucellosis in agricultural regions is suggestive of a shift of geographic distribution. The pooled prevalence of brucellosis is higher in goat flocks than in sheep flocks in China.

**Conclusions:**

The overall data in this meta-analysis demands comprehensive intervention measures and further surveillance to facilitate the control of brucellosis in livestock. Further studies aimed at evaluating the risk factors associated with spreads of brucellosis in domestic animals unaddressed so far, and sufficient epidemiological data is important to the exploration and understanding of the prevalent status of brucellosis throughout the country and to disease control.

**Electronic supplementary material:**

The online version of this article (10.1186/s12917-018-1715-6) contains supplementary material, which is available to authorized users.

## Background

Brucellosis is a highly contagious zoonotic disease caused by various species of the genus *Brucella* and poses a threat to public health; over half a million cases of the disease are reported annually [1, 2]. Meanwhile, brucellosis causes significant economic losses to the animal industry worldwide because it usually results in abortion, infertility and decrease in milk and meat production [3]. Brucellosis is effectively controlled in developed countries, [4, 5], but it remains endemic in some developing countries and regions, especially in Middle East [6, 7], Africa [8–10], Central America [11] or Latin America [12] and Asia including China [13–15], where the seroprevalence and incidence of human brucellosis are also increasing or even highly prevalent despite great efforts for the prevention and control of disease [16].

To date, brucellosis has been reported in 30 of the 32 provinces or autonomous regions of China [17, 18], where northeast China and northwest China appear to be severely afflicted by brucellosis that affects humans [19, 20] and domestic animals [21, 22]. In the last decade, the increasing demand of dairy products, including goat milk and corresponding material for industrial production, and the blind expansion of farming scale, have promoted investments in domestic animal ranching in mainland China and have dramatically increased the frequency of transport of breeding animals, which may have accelerated the spread of *Brucella* and increased the prevalence of brucellosis. Thus, humans, especially herdsmen and veterinarians, are at increased risk of being exposed to *Brucella* [23–25]. Additionally, a study has confirmed that 79.4% of the patients with brucellosis had histories of having close contact with domestic animals [26]. During the past decade, new cases of human brucellosis have been reported, and the disease had a dramatic geographic expansion from Northern China [27]. Furthermore, non-occupational exposure may have been common because of the easy movement of animals and acquirement of animal food from brucellosis-endemic regions. Presently, brucellosis in domestic animals is the major cause of human infection. Thus, a substantial decline in the incidence rate of human brucellosis is expected when the prevalence of brucellosis is controlled by eliminating positive-animal reservoirs [1].

Human brucellosis is mainly caused by exposure to *Brucella*-infected livestock, aborted materials or their products or by consuming unpasteurized food contaminated by *Brucella* spp*.*, especially milk or milk products of sheep and goats [28]. Moreover, the high incidence of human brucellosis is associated with the high density of sheep and goats and not with the high density of swine and cattle [29]. Among the nine known *Brucella* species, *B. melitensis* is the most virulent and invasive [30]. The epidemiological studies revealed that 84.5% of the 634 strains isolated from the patients with brucellosis are *B. melitensis* [31]. Therefore, the prevention and control of brucellosis in small ruminants will contribute to decline of human brucellosis incidence, especially in the endemic regions of China. However, epidemiological data is the first prerequisite for the implementation of a comprehensive campaign aimed at controlling brucellosis throughout the country. To the best of our knowledge, no study has estimated the seroprevalence of brucellosis in ovine and caprine flocks in China. Therefore, we conducted this systematic review and meta-analysis to evaluate the incidence of brucellosis in ovine and caprine herds in China. Our study may facilitate the prevention and control of the diseases associated with various species of *Brucella*.

## Results

### Studies included

**Fig. 1:**
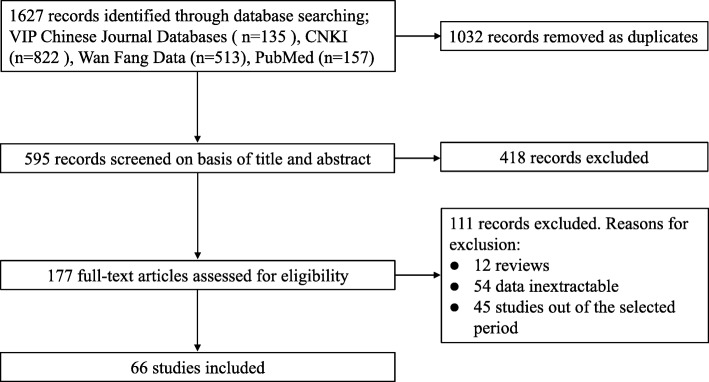
Flow diagram of literature search and selection

A total of 1627 relevant articles related to *Brucella* infection were retrieved, from which 66 articles were selected for quantitative analysis (Fig. 1 and Additional file 1). Then, 51 papers were excluded because the species in the studies were not described. The quality of the articles was evaluated according to the following criteria: content of articles, prevalence of brucellosis, purpose of research, and comprehensiveness of data presented in the selected studies, 25 papers were scored to be high quality (3 or 4 points), 20 scored medium quality (2 points), and the 21 papers were classified into the low quality (0 or 1 point) (Additional file 1).

### Publication Bias

The extent of publication bias in the selected studies was measured and demonstrated by the funnel plot (Additional file 1, Figs. 2 and 3, respectively). The medium asymmetry was confirmed. Each of the selected paper had a slight publication bias, which may have likely affected the analysis (Table 1).

**Fig. 2:**
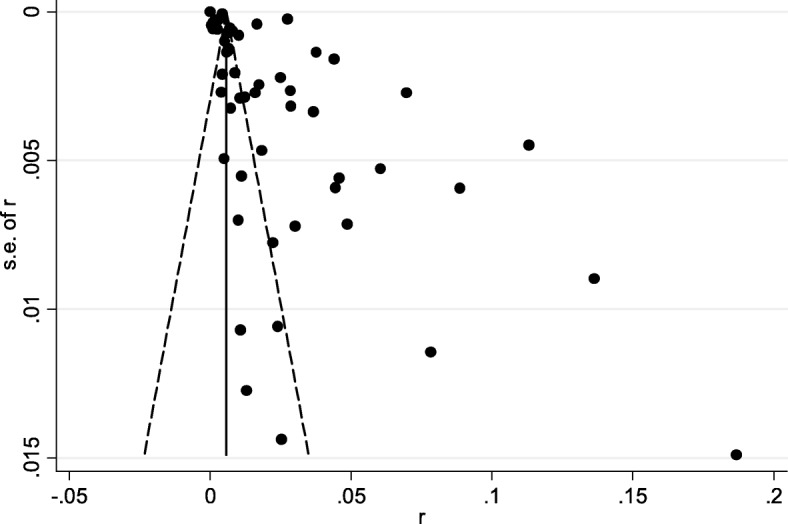
Funnel plot with pseudo 95% confidence limits for examination of publication bias

**Fig. 3:**
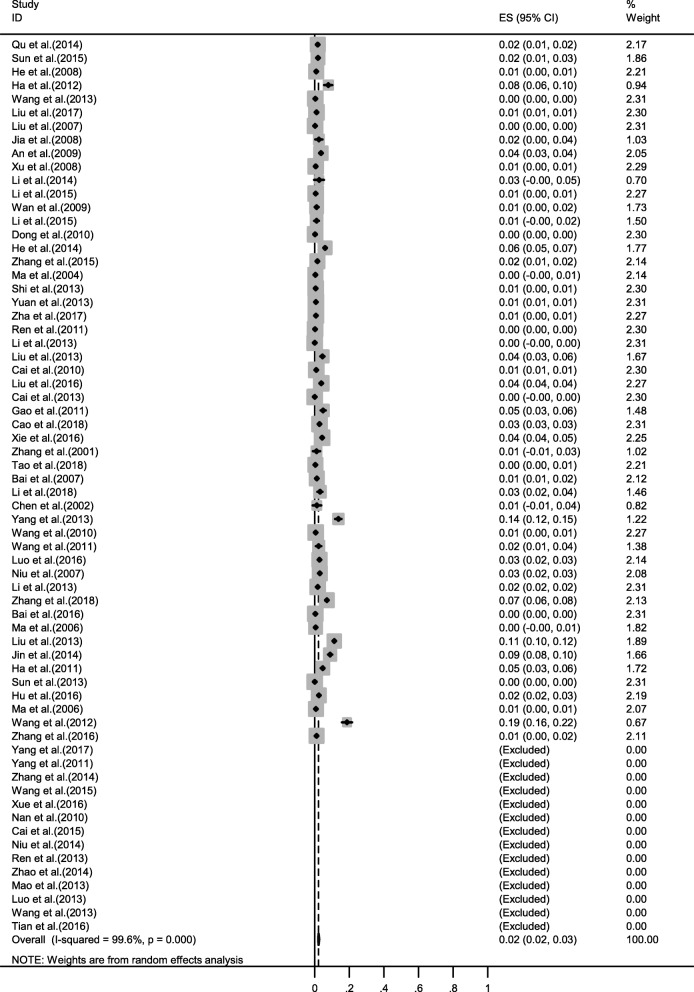
Forest plot of prevalence of brucellosis in ovine and caprine flocks amongst studies conducted in China

**Table 1:** Association of different variables in the seroprevalence of brucellosis in ovine and caprine in China

	Variables	No. studies	No. samples	No. Positive	Rate (%) (95% CI)	Heterogeneity			Regression analysis	
						χ^2^	*P*-value	I^2^ (%)	(95% CI)	*P*-value
Region	Northeast China*	2	2651	211	5.60 (0–12.10)	46.10	0.000	97.8%	0.0001(0.0000–0.0084)	0.000
	Northern China	3	846,759	3663	3.20 (0–6.60)	84.81	0.000	97.6%		
	Northwest China	42	644,113	13,592	1.40 (1.00–1.80)	8545.14	0.000	99.6%		
	Eastern China	4	1066	130	7.00 (0–17.40)	106.33	0.000	98.1%		
	Southern China	3	6296	129	2.00 (0.50–3.60)	17.10	0.000	94.2%		
	Central China	2	1796	30	1.90 (0–3.80)	6.39	0.012	84.3%		
Sampling year	Southwest China	10	132,061	3814	5.30 (3.60–7.10)	1286.40	0.000	99.5%	0.0001(0.0000–0.0001)	0.000
	2010–2018	47	1,474,801	19,540	3.20 (2.70–3.60)	11,402.65	0.000	99.7%		
	2000–2009	24	159,941	2029	1.00 (0.70–1.30)	1367.24	0.000	98.4%		
Method	SAT	48	1,607,691	21,115	2.20 (1.90–2.50)	12,495.11	0.000	99.7%	0.0001(0.0001–0.0002)	0.000
	RBPT	18	27,051	454	2.80 (1.90–3.80)	219.96	0.000	95.5%		
Species	Goat	28	188,687	4246	3.50 (2.80–4.10)	2835.30	0.000	99.3%	0.0001(0.0001–0.0002)	0.000
	Sheep	45	1,446,055	17,323	1.80 (1.50–2.20)	9705.32	0.000	99.6%		
	Total	66	1,634,742	21,569	2.30 (2.00–2.60)	12,732.79	0.000	99.6%	0.0001	0.000

*CI* Confidence interval, *SAT* Serum agglutination test, *RBPT* Rose Bengal plate test

*Northeast China: Heilongjiang, Jilin, Liaoning; Northern China: Inner Mongolia, Shanxi, Hebei, Beijing, Tianjin; Northwest China: Xinjiang, Qinghai, Gansu, Ningxia, Shaanxi; Eastern China: Shandong, Anhui, Jiangxi, Jiangsu, Zhejiang, Shanghai, Fujian; Southern China: Guangxi, Guangdong, Shenzhen, Hainan, Macao, Hong Kong; Central China: Henan, Hunan, Hubei; Southwest China: Tibet, Yunnan, Guizhou, Sichuan, Chongqing

### Pooled seroprevalence of brucellosis in ovine and caprine in China

Our systematic review and meta-analysis based on 66 studies with 1,634,742 clinical samples demonstrated that seroprevalence of brucellosis in ovine and caprine flocks at the country level was 2.30% (95% CI 2.00–2.60) from 2000 to 2018 when the samples were harvested in various studies (Table 1). The prevalence rate of brucellosis between 2000 and 2009 was 1.00% (95% CI 0.70–1.30). Meanwhile, between 2010 and 2018, the seroprevalence reached 3.20% (95% CI 2.70–3.60), demonstrating significantly increasing infection by *Brucella* spp. in ovine and caprine flocks in China (*P* < 0.001).

### Pooled brucellosis seroprevalence in administrative districts or provinces of China

We aimed to evaluate the seroprevalence of *Brucella* spp. and distribution of brucellosis in China. However, most studies or sample origins were focused on northwest (42/66) and southwest China (10/66), where the populations of sheep and goats accounts for an overwhelming majority compared with the populations in other regions in China. The distribution of the samples covered 16 provinces or the regions of 32 provinces of China. Epidemiological data regarding the seroprevalence of brucellosis in other provinces are currently unavailable because the distribution of ovine and caprine herds in China is unbalanced during decades.

Our analysis demonstrated that the pooled brucellosis positive rate in ovine and caprine flocks in Eastern China was 7.00% (95% CI 0–17.40%), higher than that in other regions of China (Table 1). Comparatively, the regions where the positive rate of brucellosis was higher than 5.00% included Northeast China and Southwest China (5.60 and 5.30%, respectively). At the province level, the seroprevalence in Shandong province was the highest (18.70, 95% CI 15.80–21.60%), followed by Jilin, Guizhou and Yunnan (8.90, 5.80 and 5.30%, respectively; Table 2 and Additional file 1 Fig. 4).

**Table 2:** Estimated pooled seroprevalence of brucellosis in ovine and caprine by provincial regions in China

Province	Region	No. tested	No. positive	Rate (%)	95% CI
Liaoning	Northeast China	360	8	2.20	0.70–3.70
Jilin	Northeast China	2291	203	8.90	7.70–10.00
Inner Mongolia	Northern China	846,759	3663	3.20	0–6.60
Gansu	Northwest China	432,433	11,697	2.40	0.80–4.10
Shaanxi	Northwest China	43,443	131	0.30	0.20–0.40
Ningxia	Northwest China	4000	0	0	–
Xinjiang	Northwest China	75,658	1292	2.40	1.60–3.20
Qinghai	Northwest China	88,579	472	0.60	0.40–0.80
Henan	Central China	1796	30	1.90	0–3.80
Shandong	Eastern China	685	128	18.70	15.80–21.60
Anhui	Eastern China	93	1	1.10	0–3.20
Fujian	Eastern China	288	1	1.30	0–3.80
Yunnan	Southwest China	98,895	2160	5.30	0.70–9.80
Guizhou	Southwest China	16,639	928	5.80	2.20–9.30
Guangxi	Southern China	6296	129	2.00	0.50–3.60
Chongqing	Southern China	16,527	726	4.40	4.10–4.70

*CI* Confidence interval

**Fig. 4:**
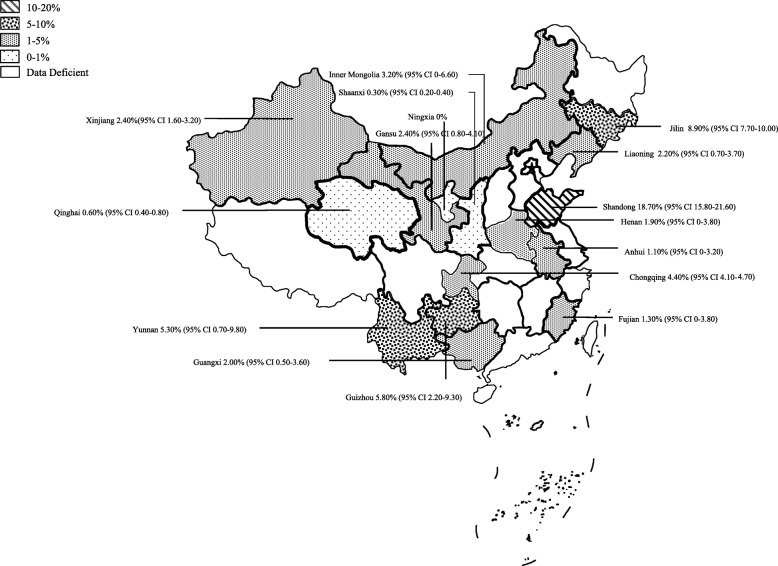
Map of prevalence of brucellosis in ovine and caprine flocks in China

### Brucellosis seroprevalence based on various of diagnostic tests

The majority of studies included in this systematic review and meta-analysis evaluated positive rate by using SAT (48/66) with pooled 2.20% seroprevalence during 2000–2018, and 18 of the 66 studies conducted assessment by using RBPT and pooled 2.80% positive rate during the period.

### Seroprevalence of brucellosis in ovine or caprine flocks

The seroprevalence of brucellosis was significantly higher in goats than in sheep (3.50% vs. 1.80%, *P* < 0.001). Sheep is the predominant breeding species in some regions, especially in Inner Mongolia, which has the largest sheep population in China (18.2% of flocks) [32]. In this meta-analysis, the number of samples from sheep was approximately 8 times of that of goats (Table 1 and Additional file 1), and the number of samples from sheep of Inner Mongolia accounted for more than half of the total samples tested (Table 1 and Additional file 1).

## Discussion

Brucellosis is one of the most widespread zoonoses globally, especially in undeveloped countries and regions, and causes significant social and economic burden to humans or livestock industry annually [33]. Brucellosis in domestic animals is the major source of human infection, and high incidence of human brucellosis is associated with high density of sheep and goats rather than of swine and cattle [29]. Therefore, detailed knowledge of the epidemiological status of brucellosis in sheep and goats has become crucial to the assessment of effective prevention measures against brucellosis in human or domestic animals. To the best of our knowledge, the study is the first to report the seroprevalence of brucellosis in ovine and caprine flocks in China.

In this systematic review and meta-analysis, we demonstrated that Eastern China has the highest seroprevalence of brucellosis in sheep and goat flocks. This region is considered a Type II general epidemic region implicated by brucellosis. Thus, a shift in the geographic distribution of brucellosis occurred from the traditional pastoral regions of Northern China to agricultural or semi-agricultural regions. In Eastern China, sheep and goat flocks in Shandong province represented the highest seroprevalence of the diseases (18.70%) [34]. Only one study regarding the prevalence of brucellosis that included in this analysis was conducted in Shandong province during the selected period. In that study, the number of sheep and goats was 685 (sheep 315 and goats 370, respectively) (Additional file 1). Moreover, the seropositive rate in the local area reached 32.3%, and the overall seropositive rates were 27.6 and 11.1% in sheep and goat flocks, respectively. Our systematic review indicates that brucellosis is highly endemic in some local regions, and thus a comprehensive surveillance of wide geographic regions is required for understanding the overall seroprevalence of brucellosis in domestic animals in this region. Livestock vaccination in endemic regions might be effective in controlling brucellosis according to the National Mid-Term and Long-Term Animal Disease Control Plan of China (2012–2020) [35]. Meanwhile, throughout China, the pooled seroprevalence of brucellosis was lower in sheep herds than in goat flocks (1.80% vs. 3.50%; Table 1), though the seroprevalence of brucellosis in sheep flocks was higher in some local areas. Some epidemiological surveys demonstrated that the prevalence of the disease was higher in goat flocks than in sheep herds in some countries [36–38]. Furthermore, the data represented that the prevalence in flocks with sheep and goats was two times higher than flocks with sheep or goats alone [36]. Given that caprine brucellosis is a neglected disease in some countries, including China, especially in agricultural regions, breeding ovine is exclusively predominant in some regions or local areas in China. However, caprine brucellosis might pose a significant threat to public health and animal industry.

In China, some indigenous sheep or goat species are the exclusive species of livestock breeding for local ranches or farms, especially in Xinjiang and Qinghai provinces of China. Species related to brucellosis prevalence monitored in numerous studies were not elucidated, and thus these papers were excluded. Therefore, the authentic incidence of brucellosis might be underestimated in some regions. Moreover, sheep populations are overwhelmingly predominant in major pasturing areas in China similar to Inner Mongolia, Xinjiang and Qinghai provinces, which are categorized as Type I brucellosis severe epidemic regions in China. In these regions, most human brucellosis cases were transmitted by sheep-type *Brucella*, and the dominant strain was *B. melitensis*. Epidemiological data revealed that 84.5% of *Brucella* strains isolated from the patients with brucellosis in China are *B. melitensis* [31]. From 1996 to 2010, 90.25% or more of the total 78,246 human brucellosis cases were caused by sheep-originated *Brucella* [21]. Furthermore, epidemiological studies confirmed that 99% of patients in Italy were infected by *B. melitensis* [30], indicating that sheep with brucellosis pose much more threat to humans than other species. Therefore, the prevention and control of brucellosis in sheep flocks are expected to reduce the incidence of human brucellosis in endemic areas, including Inner Mongolia of China, which accounts for 47.2% of new cases of human brucellosis in China in 2010 [21].

Additionally, Heilongjiang province was considered a Type I brucellosis severe epidemic region in Northern China besides Inner Mongolia and Jilin province. The incidence of human brucellosis in Heilongjiang province was 5.92 per 100,000 population in 2004, although the annual incidence of human brucellosis dramatically reached 19.45 per 100,000 population in 2012, which is the second highest after Inner Mongolia in China [20]. Notwithstanding the high prevalence of human brucellosis in Heilongjiang province of China and the importance of sheep and goat reservoirs to the spread of brucellosis, epidemiological data on the seroprevalence of brucellosis in sheep and goats in Heilongjiang province is deficient, suggesting that comprehensive epidemiological surveillance combined with intervention measures in domestic animals, especially in sheep and goat flocks, is necessary for disease control. Additionally, the milk goat industry in Heilongjiang province has been developing, and thus the comprehensive surveillance of disease in domestic animals and humans, especially diseases associated with occupational exposure, has become increasingly important.

The systematic review revealed that the selected studies for brucellosis surveillance in ovine and caprine flocks were implemented with SAT or RBPT, both of which have lower specificity and sensitivity compared with the methods recommended by WHO (indirect ELISA and fluorescent polarization assay) [39, 40]. The pooled seroprevalence based on our meta-analysis may not be consistent with the authentic incidence of brucellosis in some regions or provinces because of the limitation in the serological test used in the selected studies and agglutination tests, including SAT and RBPT, are commonly used for diagnosing acute brucellosis, which are not suitable for chronic brucellosis and infection caused by *B. canis* [41]. Therefore, the seroprevalence of brucellosis in sheep and goats in the selected regions might have been underestimated. However, we believe that this meta-analysis presented the status and tendency of brucellosis in ovine and caprine flocks in China during the periods.

Our meta-analysis has several limitations. First, there are chances that not all the publications related to sheep and goat brucellosis were included during document retrieval from the selected databases, although numerous searching MeSHs were used, partially because of the keyword selection in publication itself. Second, the risk factors associated with the incidence of brucellosis widespread of *Brucella* spp*.* were unavailable in the majority of the selected papers. Thus, the associated risk factors involved in brucellosis in sheep or goat flocks in China can not be further analyzed in this study.

## Conclusion

We conducted a systematic review and meta-analysis to assess the prevalence of brucellosis in ovine and caprine flocks in China. The prevalence of brucellosis in ovine and caprine flocks in China shows a tendency to rise and highly endemic in some regions in China. The higher prevalence of brucellosis in agricultural regions is suggestive of a shift of geographic distribution. The pooled prevalence of brucellosis is higher in goat flocks than in sheep in China. The overall data demands intervention measures, including vaccination and enhanced public awareness, and further surveillance for the control of brucellosis in livestock. Further studies that are aimed at evaluating the risk factors associated with the spread of brucellosis in domestic animals and sufficient epidemiological data are crucial to the exploration of the epidemiology of the disease throughout the country.

## Methods

### Search strategy

We performed a systematic search across four electronic databases: VIP Chinese Journal Databases, China National Knowledge Infrastructure (CNKI), Wan Fang Data and PubMed with the following Mesh terms and key word subject heading “brucellosis”, "*Brucella*" or their various short terms in Chinese, or synonymous terms “brucellosis” and synonymous terms of brucellosis, “Malta fever”, “Mediterranean fever”, “Mediterranean remittent fever”, “Undulant fever”, “Gibraltar fever”, “Rock fever” or “Neapolitan fever” and “seroprevalence”, “prevalence”, “surveillance” “epidemiological survey”, “sheep or ovine”, “goat or caprine” and “China” were included during searching in PubMed. We focused on studies about the brucellosis seroprevalence of natural infection in ovine and caprine flocks in China. The samples in these studies were collected from January 1, 2000 to June 1, 2018. The reviews, duplicate reports, studies for other species (e.g., human, cattle, swine, bison, dog, water buffalo, yak, deer and takins) and evaluation of vaccine efficacy in herds were excluded, as well as studies and reports that only reported seroprevalence without primary data, had sample size of < 30, included regions out of China and modelling studies. Additionally, the studies in which the diagnostic methods or species were not clearly described was also excluded from this analysis.

### Literature screening and data extraction

Reviewers independently extracted and recorded data from each selected study. Any disagreement between the reviewers or uncertainty about the eligibility of a study was further evaluated by additional reviewers. Information was recorded as follows: the first author, the year of sample isolation, year of publication, location of flocks, diagnostic tests, the number of tested sheep or goats, and the number of seropositive animals. Moreover, we neither contacted the authors of the original studies for additional information nor identified unpublished data. The data collection form that was used for this analysis is presented in Additional file 1.

### Quality assessment

The quality of the eligible publications was estimated according to the criteria derived from the Grading of Recommendations Assessment, Development and Evaluation method. The quality of the publications was graded by using a scoring approach. Briefly, the score for each of the following items was determined as 1 point when information was elaborated: object, detection method used, sampling time and classification of a subgroup in a study. Papers would be assigned 0–4 points on the basis of score criterion. Studies with 3–4 points were considered high quality, those with 2 points were deemed moderate and those with scores of 0–1 point was designated as low quality.

### Statistical analysis

The pooled seroprevalence of brucellosis in ovine and caprine based on publications by numerous studies were calculated by meta-analysis. Heterogeneity in the qualified studies was expected, and thus a random-effects model was used for the calculation and preparation of the forest plots with the code of “generate ser=sqrt(r*(1-r)/n); metan r ser, random label (namevar=study)” with Stata 12 (Stata Corp. College station, Texas). The heterogeneity was anticipated in advance, and statistical methods with I^2^ and Cochrane Q (represented as χ^2^ and *P* values) statistics were used for the assessment of the variations. The potential sources of heterogeneity were further investigated by subgroup and meta-regression analyses. The factors associated with heterogeneity in this study were examined on the basis of an individual model or multiple-variable models. Basically, the factors include sampling year (comparison between sample harvesting time of before 2010 [2000–2009] with that of 2010 or later [2010–2018]), administrative districts or regions, diagnostic methods (serum agglutination test [SAT] VS. Rose Bengal plate test [RBPT]). The meta-analysis was performed according to the PRISMA guideline [42] and the confidence intervals of the seroprevalence of brucellosis were calculated using Woolf’s method with Stata program.

## Additional file

Additional file 1: Included studies of brucellosis prevalence in sheep and goats in China. (DOC 312 kb)

## Abbreviations

ELISA: Enzyme-linked immunosorbent assay
RBPT: Rose Bengal plate test
SAT: Serum agglutination test
