# Correction to: Brucellosis seroprevalence in ovine and caprine flocks in China during 2000–2018: a systematic review and meta-analysis

**DOI:** 10.1186/s12917-018-1749-9

**Published:** 2019-01-08

**Authors:** Xuhua Ran, Xiaohong Chen, Miaomiao Wang, Jiajia Cheng, Hongbo Ni, Xiao-Xuan Zhang, Xiaobo Wen

**Affiliations:** College of Animal Science & Veterinary Medicine, Heilongjiang Bayi Agricultural University, No.5, XinFeng Rd., Saertu District, Daqing City, 163319 Heilongjiang Province China

**Correction to: BMC Vet Res**

**https://doi.org/10.1186/s12917-018-1715-6**

The original article [1] contained a minor error whereby Table 1 was typeset incorrectly. This error has now been corrected.
