# Supplementary material for: Brucellosis seroprevalence in ovine and caprine flocks in China during 2000–2018: a systematic review and meta-analysis
Source: BMC Vet Res. 2018 Dec 12;14:393. doi: 10.1186/s12917-018-1715-6 (PMC6292006; doi:10.1186/s12917-018-1715-6)
Supplement: Supplementary file 1 — Included studies of brucellosis prevalence in sheep and goats in China. (DOC 312 kb) [file 12917_2018_1715_MOESM1_ESM.doc]

**Additional file 1** Included studies of brucellosis prevalence in sheep and goats in China.

| Study ID | Province/region | Region | Species | Sampling  years | No.  tested | No. positive | Prevalence  % | Detection method | Study design | Quality score |
| --- | --- | --- | --- | --- | --- | --- | --- | --- | --- | --- |
| Wang et al.(2011) | Liaoning | Northeast China | Goat | 2010 or later | 360 | 8 | 2.22 | RBPT | Cross sectional | 1 |
| Jin et al.(2014) | Jilin | Northeast China | Goat/Sheep | 2010 or later | 2291 | 203 | 8.86 | SAT | Cross sectional | 3 |
| Wang et al.(2013) | Inner Mongolia | Northern China | Sheep | 2010 or later | 844637 | 3565 | 0.42 | SAT | Cross sectional | 3 |
| Liu et al.(2013) | Inner Mongolia | Northern China | Sheep | 2010 or later | 1216 | 54 | 4.44 | RBPT | Cross sectional | 1 |
| Gao et al.(2011) | Inner Mongolia | Northern China | Sheep | Before 2010 | 906 | 44 | 4.86 | RBPT | Cross sectional | 1 |
| Qu et al.(2014) | Xinjiang | Northwest China | Sheep | Before 2010 | 1255 | 16 | 1.27 | SAT | Cross sectional | 4 |
| Qu et al.(2014) | Xinjiang | Northwest China | Sheep | 2010 or later | 1564 | 33 | 2.11 | SAT | Cross sectional | 4 |
| Ha et al.(2012) | Xinjiang | Northwest China | Sheep | 2010 or later | 550 | 43 | 7.82 | RBPT | Cross sectional | 2 |
| Liu et al.(2017) | Xinjiang | Northwest China | Sheep | 2010 or later | 16458 | 168 | 1.02 | SAT | Cross sectional | 4 |
| Shi et al.(2013) | Xinjiang | Northwest China | Sheep | 2010 or later | 12761 | 80 | 0.63 | RBPT | Cross sectional | 3 |
| Yuan et al.(2013) | Xinjiang | Northwest China | Sheep | 2010 or later | 22213 | 154 | 0.69 | SAT | Cross sectional | 4 |
| Liu et al.(2016) | Xinjiang | Northwest China | Sheep | 2010 or later | 19460 | 734 | 3.77 | SAT | Cross sectional | 2 |
| Ha et al.(2011) | Xinjiang | Northwest China | Goat/Sheep | 2010 or later | 1397 | 64 | 4.58 | RBPT | Cross sectional | 2 |
| Yang et al.(2017) | Qinghai | Northwest China | Sheep | 2010 or later | 538 | 0 | 0 | SAT | Cross sectional | 4 |
| He et al.(2008) | Qinghai | Northwest China | Sheep | Before 2010 | 2058 | 18 | 0.87 | RBPT | Cross sectional | 3 |
| Yang et al.(2011) | Qinghai | Northwest China | Sheep | Before 2010 | 2051 | 0 | 0 | SAT | Cross sectional | 2 |
| Yang et al.(2011) | Qinghai | Northwest China | Sheep | 2010 or later | 300 | 0 | 0 | SAT | Cross sectional | 2 |
| Zhang et al.(2014) | Qinghai | Northwest China | Sheep | 2010 or later | 538 | 0 | 0 | SAT | Cross sectional | 2 |
| Liu et al.(2007) | Qinghai | Northwest China | Sheep | Before 2010 | 22008 | 40 | 0.18 | SAT | Cross sectional | 1 |
| Jia et al.(2008) | Qinghai | Northwest China | Sheep | Before 2010 | 209 | 5 | 2.39 | SAT | Cross sectional | 1 |
| Wang et al.(2015) | Qinghai | Northwest China | Sheep | 2010 or later | 200 | 0 | 0 | RBPT | Cross sectional | 1 |
| Xue et al.(2016) | Qinghai | Northwest China | Sheep | 2010 or later | 198 | 0 | 0 | RBPT | Cross sectional | 1 |
| An et al.(2009) | Qinghai | Northwest China | Sheep | Before 2010 | 3111 | 114 | 3.66 | SAT | Cross sectional | 1 |
| Xu et al.(2008) | Qinghai | Northwest China | Sheep | Before 2010 | 5206 | 27 | 0.52 | SAT | Cross sectional | 2 |
| Li et al.(2014) | Qinghai | Northwest China | Sheep | 2010 or later | 119 | 3 | 2.52 | RBPT | Cross sectional | 2 |
| Li et al.(2015) | Qinghai | Northwest China | Sheep | 2010 or later | 3100 | 18 | 0.58 | SAT | Cross sectional | 2 |
| Nan et al.(2010) | Qinghai | Northwest China | Sheep | 2010 or later | 262 | 0 | 0 | RBPT | Cross sectional | 1 |
| Wan et al.(2009) | Qinghai | Northwest China | Sheep | Before 2010 | 360 | 4 | 1.11 | SAT | Cross sectional | 1 |
| Li et al.(2015) | Qinghai | Northwest China | Sheep | 2010 or later | 201 | 2 | 1.00 | RBPT | Cross sectional | 1 |
| Cai et al.(2015) | Qinghai | Northwest China | Sheep | 2010 or later | 204 | 0 | 0 | RBPT | Cross sectional | 1 |
| Dong et al.(2010) | Qinghai | Northwest China | Sheep | Before 2010 | 6932 | 17 | 0.25 | SAT | Cross sectional | 2 |
| Zha et al.(2017) | Qinghai | Northwest China | Sheep | 2010 or later | 4119 | 26 | 0.63 | SAT | Cross sectional | 3 |
| Ma et al.(2004) | Qinghai | Northwest China | Sheep | Before 2010 | 520 | 2 | 0.38 | SAT | Cross sectional | 1 |
| Ren et al.(2011) | Qinghai | Northwest China | Sheep | Before 2010 | 6932 | 17 | 0.25 | SAT | Cross sectional | 1 |
| Ren et al.(2013) | Qinghai | Northwest China | Sheep | 2010 or later | 1219 | 0 | 0 | SAT | Cross sectional | 1 |
| Li et al.(2013) | Qinghai | Northwest China | Sheep | Before 2010 | 467 | 1 | 0.21 | SAT | Cross sectional | 1 |
| Li et al.(2013) | Qinghai | Northwest China | Sheep | 2010 or later | 1793 | 0 | 0 | SAT | Cross sectional | 1 |
| Cai et al.(2010) | Qinghai | Northwest China | Sheep | Before 2010 | 18320 | 147 | 0.80 | SAT | Cross sectional | 1 |
| Cai et al.(2013) | Qinghai | Northwest China | Sheep | Before 2010 | 3000 | 3 | 0.1 | SAT | Cross sectional | 1 |
| Ma et al.(2006) | Qinghai | Northwest China | Goat | Before 2010 | 202 | 1 | 0.50 | SAT | Cross sectional | 1 |
| Ma et al.(2006) | Qinghai | Northwest China | Goat/Sheep | Before 2010 | 689 | 5 | 0.73 | SAT | Cross sectional | 1 |
| Wang et al.(2010) | Qinghai | Northwest China | Goat | Before 2010 | 3512 | 22 | 0.63 | SAT | Cross sectional | 1 |
| Wang et al.(2013) | Qinghai | Northwest China | Goat | 2010 or later | 211 | 0 | 0 | SAT | Cross sectional | 2 |
| Bai et al.(2016) | Shaanxi | Northwest China | Goat | Before 2010 | 12407 | 21 | 0.17 | SAT | Cross sectional | 2 |
| Bai et al.(2016) | Shaanxi | Northwest China | Goat | 2010 or later | 31036 | 110 | 0.35 | SAT | Cross sectional | 2 |
| Niu et al.(2014) | Ningxia | Northwest China | Sheep | 2010 or later | 4000 | 0 | 0 | SAT | Cross sectional | 2 |
| Cao et al.(2018) | Gansu | Northwest China | Sheep | 2010 or later | 419606 | 11518 | 2.74 | SAT | Cross sectional | 3 |
| He et al.(2014) | Gansu | Northwest China | Sheep | 2010 or later | 2036 | 123 | 6.04 | SAT | Cross sectional | 3 |
| Zhang et al.(2015) | Gansu | Northwest China | Sheep | 2010 or later | 2084 | 33 | 1.58 | SAT | Cross sectional | 2 |
| Sun et al.(2015) | Gansu | Northwest China | Sheep | 2010 or later | 821 | 15 | 1.83 | RBPT | Cross sectional | 3 |
| Sun et al.(2013) | Gansu | Northwest China | Goat/Sheep | 2010 or later | 7886 | 8 | 0.1 | SAT | Cross sectional | 4 |
| Zhang et al.(2001) | Anhui | Eastern China | Goat | Before 2010 | 93 | 1 | 1.08 | SAT | Cross sectional | 2 |
| Mao et al.(2013) | Fujian | Eastern China | Goat | 2010 or later | 210 | 0 | 0 | RBPT | Cross sectional | 3 |
| Chen et al.(2002) | Fujian | Eastern China | Goat | Before 2010 | 78 | 1 | 1.228 | SAT | Cross sectional | 2 |
| Wang et al.(2012) | Shandong | Eastern China | Goat/Sheep | 2010 or later | 685 | 128 | 18.69 | SAT | Cross sectional | 3 |
| Zhao et al.(2014) | Guangxi | Southern China | Goat | 2010 or later | 924 | 0 | 0 | SAT | Cross sectional | 4 |
| Bai et al.(2007) | Guangxi | Southern China | Goat | Before 2010 | 1469 | 18 | 1.23 | SAT | Cross sectional | 3 |
| Luo et al.(2016) | Guangxi | Southern China | Goat | 2010 or later | 3903 | 111 | 2.84 | SAT | Cross sectional | 4 |
| Li et al.(2018) | Henan | Central China | Goat | 2010 or later | 563 | 17 | 3.02 | SAT | Cross sectional | 3 |
| Zhang et al.(2016) | Henan | Central China | Goat/Sheep | 2010 or later | 1233 | 13 | 1.05 | SAT | Cross sectional | 4 |
| Xie et al.(2016) | Chongqing | Southwest China | Goat | 2010 or later | 16527 | 726 | 4.39 | SAT | Cross sectional | 2 |
| Tao et al.(2018) | Guizhou | Southwest China | Goat | 2010 or later | 950 | 4 | 0.42 | SAT | Cross sectional | 4 |
| Luo et al.(2013) | Guizhou | Southwest China | Goat | 2010 or later | 514 | 0 | 0 | RBPT | Cross sectional | 2 |
| Yang et al.(2013) | Guizhou | Southwest China | Goat | 2010 or later | 1461 | 199 | 13.62 | SAT | Cross sectional | 2 |
| Tian et al.(2016) | Guizhou | Southwest China | Goat | 2010 or later | 126 | 0 | 0 | RBPT | Cross sectional | 3 |
| Zhang et al.(2018) | Guizhou | Southwest China | Goat | 2010 or later | 8640 | 602 | 6.97 | SAT | Cross sectional | 3 |
| Hu et al.(2016) | Guizhou | Southwest China | Goat/Sheep | 2010 or later | 4948 | 123 | 2.49 | RBPT | Cross sectional | 3 |
| Niu et al.(2007) | Yunnan | Southwest China | Goat | Before 2010 | 2756 | 79 | 2.87 | SAT | Cross sectional | 2 |
| Li et al.(2013) | Yunnan | Southwest China | Goat | Before 2010 | 65400 | 1426 | 2.18 | SAT | Cross sectional | 2 |
| Li et al.(2013) | Yunnan | Southwest China | Goat | 2010 or later | 25737 | 89 | 0.35 | SAT | Cross sectional | 2 |
| Liu et al.(2013) | Yunnan | Southwest China | Goat | 2010 or later | 5002 | 566 | 11.32 | SAT | Cross sectional | 3 |

SAT: Serum agglutination test; RBPT: Rose Bengal plate test

**Reference**

1. Wang M. Serological detection of brucellosis, chlamydiosis and toxoplasmosis in cashmere goat. Journal of Domestic Animal Ecology 2011; 32(6):80-1+123 (In Chinese).

2. Jin C. Epidemiological investigation on brucellosis in some areas of Jilin province from 2010 to 2013. Jilin Animal Husbandry and Veterinary Medicine 2014; 35(9):26-8 (In Chinese).

3. Wang J, Wang Y, Liu L, Guo H, Feng L. Epidemiological investigation and analysis of sheep brucellosis in Aohanqi, Inner Mongolia. Animal Husbandry and Feed Science 2013; 34(11):103-4 (In Chinese).

4. Liu X, Gao Y. Serological investigation on serum antibody of sheep brucellosis in Ximeng East Soviet Banner in Inner Mongolia. Animal Husbandry and Veterinary Science & Technology information 2013; (12):20-1 (In Chinese).

5. Gao M. Serologic investigation of *Brucella* infection in sheep in Hulunbiur. Animal Husbandry and Feed Science 2011; 3(6-12):21-2.

6. Qu M. Epidemiological survey and analysis of prevention and control status of brucellosis in cattle in 181 groups of Xinjiang. Master's Thesis*.* Shihezi University; 2014 (In Chinese).

7. Haxi B, Xin N, Cairen J, Batu A, Ba T. Monitoring and purification of brucellosis in domestic animals in an agricultural and forestry bureau. The Chinese Livestock and Poultry Breeding 2012; 8(2):47-8 (In Chinese).

8. Liu Y, Xinhuang, Cai G, Han M. Serum epidemiology of brucellosis in cattle and sheep. Xinjiang Farm Research of Science and Technology 2017; 40(6):29-31 (In Chinese).

9. Shi Q, Yuan L, Pu J, Liang J. Investigation and analysis of Brucella infection in cattle and sheep in mountainous area. China Animal Health Inspection 2013; 30(2):40-1 (In Chinese).

10. Yuan L, Shi Q, Pu J. Investigation and analysis of brucellosis in cattle and sheep in mountain area. Animal Husbandry Veterinary Medicine 2013; 45(9):119-20 (In Chinese).

11. Liu J. Epidemiological survey and analysis of prevention on brucellosis in Baicheng County of Xinjiang. Master's Thesis. Shihezi University; 2016 (In Chinese).

12. Haxi B. Investigation on brucellosis in domestic animal in Hejing county, Bazhou. Xinjiang Animal Husbandry 2011; (11):32-3 (In Chinese).

13. Yang C, Qi Y, Lv J. Serological investigation on brucellosis, chlamydia and toxoplasmosis in Qinghai semi-fine wool sheep in Dulan county. Chinese Qinghai Journal of Animal and Veterinary Sciences 2017; 47(6):52-3 (In Chinese).

14. He S, Che F. Monitoring and control of brucellosis in cattle and sheep in area around Qinghai lake. Animal Husbandry Veterinary Medicine 2008; 40(5):109-10 (In Chinese).

15. Yang L. Monitor, investigation and control measures of brucellosis and tuberculosis in Minhe county. China Dairy Cattle 2011; (1):59-62 (In Chinese).

16. Zhang Z. Analysis of prevalence of chlamydia and brucellosis in Tibetan plateau Mongolian sheep. Jiangxi Journal of Animal Husbandry & Veterinary Medicine 2014; (5):42-3 (In Chinese).

17. Liu H, Dong C. Monitoring of brucellosis in yak and Tibetan sheep in Guluo area of Qinghai province. Chinese Journal of Veterinary Medicine 2007; 43(5):37 (In Chinese).

18. Jia S, Niu X. Serological investigation of brucellosis in the Tibetan community of Haiyan pasture, Qinghai province. Chinese Qinghai Journal of Animal and Veterinary Sciences 2008; 38(1):30 (In Chinese).

19. Wang X, Li X, Cai R, Wang G, Wang G, Ma L. Serological detection of abortion diseases in Oura-type of Tibetan sheep in Henan County of Qinghai. Chinese Qinghai Journal of Animal and Veterinary Sciences 2015; 45(4):8-10 (In Chinese).

20. Xue H, Li X, Jin L. Investigation on brucellosis in Hudong sheep farm of Qinghai province. Chinese Qinghai Journal of Animal and Veterinary Sciences 2016; 46(6):56 (In Chinese).

21. An J. Serological investigation of brucellosis in cattle and sheep in Mole town, Qilian county, Qinghai province. Animals Breeding and Feed 2010; (9):36-7 (In Chinese).

22. Xu L. Investigation and analysis of brucellosis in breeding sheep in Triangle city of Qinghai province. Qinghai Medical Journal 2008; 38(5):59-61 (In Chinese).

23. Li G, Li G, Li X, Qi Q, Li F, Pei Q et al. Serological detection of abortion diseases in breeding sheep in Triangle city of Qinghai province. Chinese Qinghai Journal of Animal and Veterinary Sciences 2014; 44(2):17-8 (In Chinese).

24. Li J, Li W, Wan M, Zhang H. Serological investigation on brucellosis inTibetan goats in Tianjun county Qinghai province. Chinese Qinghai Journal of Animal and Veterinary Sciences 2015; 45(5):35 (In Chinese).

25. Nan J, Cai Q. Serological investigation of sheep brucellosis in Tianjun county, Qinghai province. Chinese Qinghai Journal of Animal and Veterinary Sciences 2010; 40(4):20 (In Chinese).

26. Wan M, Lu Y, Zhang X. Serological investigation of brucellosis in original Tibetan sheep/goat in Tianjun county, Qinghai province. China Animal Health Inspection 2009; 26(9):51 (In Chinese).

27. Li X, Zeng B, Wang G, Wang G, Ma L. Serological detection of abortion diseases in sheep in Tongde County of Qinghai Province. Chinese Qinghai Journal of Animal and Veterinary Sciences 2015; 45(2):8-9 (In Chinese).

28. Cai W, Li X, Wang G, Ma L. Serological detection of abortion diseases in sheep in Maqin County of Qinghai Province. Chinese Qinghai Journal of Animal and Veterinary Sciences 2015; 45(4):6-7 (In Chinese).

29. Dong Y, Xu Z, Li W, Zhang X. Investigation on *Brucella* infection in livestock in Tianjun area of Qinghai province. Animal Husbandry Veterinary Medicine 2010; 42(7):108-9 (In Chinese).

30. Zha X, Xu X. Serological investigation on brucellosis in Tibetan sheep in Shengge township. The Chinese Livestock and Poultry Breeding 2017; 13(9):47 (In Chinese).

31. Ma S, Lu Y, Li X, Guanque Z, Yun S, Dang H. Serological investigation on brucellosis in breeding sheep in Triangle city of Qinghai province. Chinese Qinghai Journal of Animal and Veterinary Sciences 2004; 34(1):26 (In Chinese).

32. Ren Q, Li W, Zhang X. Serological investigation on brucellosis in Tibetan breeding ram in some villages and towns of Tianjun county. Chinese Qinghai Journal of Animal and Veterinary Sciences 2011; 41(2):35 (In Chinese).

33. Ren Q, Zhang H, Zhang X. Serological investigation of brucellosis in breeding ram in Suli town in Tianjun county. Chinese Qinghai Journal of Animal and Veterinary Sciences 2013; 43(4):23 (In Chinese).

34. Li Q, Han X, Wang S, Zhang G, Qi Y, Zhen S. Epidemiological investigation on brucellosis in cattle and sheep in Xining city. China Animal Health Inspection 2013; 30(5):36-7 (In Chinese).

35. Cai R. Detection and control of brucellosis of fine wool sheep. Chinese Livestock and Poultry Breeding 2010; 6(7):103 (In Chinese).

36. Cai R. Serological investigation on brucellosis in yak and Tibetan sheep/goat in Xinghai county. Animal Husbandry Veterinary Medicine 2013; 45(1):108 (In Chinese).

37. Ma S, Wang G. Serological investigation on brucellosis in cashmere goat in Wulan county. Chinese Qinghai Journal of Animal and Veterinary Sciences 2006; 36(1):28 (In Chinese).

38. Ma S, Ma L, Lu Y. Serological investigation of sheep brucellosis in Haixi district, Qinghai province. China Animal Health Inspection 2006; 23(1):33 (In Chinese).

39. Wang Q, Gao L, Yang K, Jiao H, Chen D. A serological investigation on etiology of abortion in sheep/goats in Haixi, Qinghai Province. Chinese Qinghai Journal of Animal and Veterinary Sciences 2010; 40(4):8 (In Chinese).

40. Wang G, Xie C, Ye C, Han Y, Wang G, Wang S et al. Investigation and prevention research on diseases associated with goat abortion in Wulan County in Qinghai Province. Chinese Qinghai Journal of Animal and Veterinary Sciences 2013; 43(3):4-6 (In Chinese).

41. Bai G, Wang G, Wang G, Wu Q. Serological investigation on brucellosis in cattle and sheep from 2008 to 2014 in Weinan city. Heilongjiang Animal Science and Veterinary Medicine 2016; (16):114-5+291 (In Chinese).

42. Niu J, Ren D, Zhang X. Diagnosis of brucellosis in sheep. China Herbivore Science 2014; (S1):346-8 (In Chinese).

43. Cao X, Li S, Li Z, Liu Z, Ma J, Lou Z et al. Enzootic situation and molecular epidemiology of *Brucella* in livestock from 2011 to 2015 in Qingyang, China. Emerging microbes & infections 2018; 7(1):58.

44. He J. Serological test on brucellosis, mycoplasma pneumonia and chlamydiosis of captive breeding sheep. Journal of Animal Science and Veterinary Medicine 2014; 33(5):19-20 (In Chinese).

45. Zhang C. Epidemic Investigation in three large-scale sheep farms and introspection. Journal of Animal Science and Veterinary Medicine 2015; 34(4):64-5 (In Chinese).

46. Sun S. Epidemiological investigation of sheep brucellosis in three counties of Gansu Province in 2014. Master's Thesis. Gansu Agricultural University; 2015 (In Chinese).

47. Sun X, Li J, Zhang S. Results and analysis of serological monitoring of brucellosis of sheep in Lanzhou. The Chinese Livestock and Poultry Breeding 2013; 9(11):28-9 (In Chinese).

48. Zhang Y. Serological investigation of brucellosis in Chizhou city. China Animal Health Inspection 2001~~(~~(8):37 (In Chinese).

49. Mao K, Lin Z, Lin X, Lin Z, Chen Y, Wu D. Serological investigation on chlamydia, toxoplasmosis and brucellosis of goats in Fuqing. Fujian Journal of Animal Husbandry and Veterinary Medicine 2013; 35(5):21-2 (In Chinese).

50. Chen Z, Chen J. Serological investigation on brucellosis of domestic animals in Putian city. Fujian Journal of Animal Husbandry and Veterinary Medicine 2002; (1):2 (In Chinese).

51. Wang Q. Seroepidemiological survey of brucellosis, foot-and-mouth disease and Hepatitis E in sheep and goats in part areas of Shandong. Master's Thesis*.* Shandong Agricultural University; 2012 (In Chinese).

52. Zhao C, Meng Z, Qin Y, Huang B. Seroepidemiological investion on brucellosis and chlamydiosis in goats in Baise City. Popular Science & Technology 2014; 16(2):85-7 (In Chinese).

53. Bai A, Liang J, Du J, Liang B, Huang A, Yao R et al. Serological investigation on brucellosis in goats in Guangxi. Guangxi Journal of Animal Husbandry & Veterinary Medicine 2007; 23(2):58-9 (In Chinese).

54. Luo Y, Huang S, Li Z. Epidemiological investigation and analysis of brucellosis in goat flocks. Contemporary Animal Husbandry 2016((21):19-21 (In Chinese).

55. Li Z, Su Y. Serological investigation of three kinds of diseases in white goat flocks in Lushan county. China Herbivore Science 2018; 38(1):47-8+56 (In Chinese).

56. Zhang G. Serological test and epidemiological investigation of sheep brucellosis in southern Henan. Heilongjiang Animal Science and Veterinary Medicine 2016; (18):135-6 (In Chinese).

57. Xie Y. Analysis on epidemiological status of brucellosis in Qijiang District of Chongqing in 2015. Occup and Heaith 2016; 32(24):3385-7 (In Chinese).

58. Tao M, Yu B, Feng Y. Serological investigation of brucellosis and O-type foot-and-mouth disease of goat in Dejiang county. Guizhou Animal Science and Veterinary Medicine 2018; 42(1):30-1 (In Chinese).

59. Luo Y, Zhou B, Zhang H, Wang K, Wen M, Cheng Z et al. Epidemiological investigation of major goat abortion epidemic in 7 districts of Guizhou province. Animal Husbandry Veterinary Medicine 2013; 45(6):62-5 (In Chinese).

60. Yang X, Wang Z, Mo X, Yang M, Huang G, Zhao X et al. Serological survey on goat brucellosis in Qiandongnan State. Guizhou Animal Science and Veterinary Medicine 2013; 37(5):7-8 (In Chinese).

61. Tian H, Wu Z, Ren Q, Yang X, Yang Q. Serological investigation and analysis of brucellosis and toxoplasmosis of white goat in Yinjiang county. Guizhou Animal Science and Veterinary Medicine 2016; 40(5):33-4 (In Chinese).

62. Zhang J, Yin S, Lu A, Bai M, Ren P, Wu C et al. Sero-epidemiological investigation on brucellosis of goat in Tongren Area. Journal of Anhui Agricultural Sciences 2018; 46(11):63-5 (In Chinese).

63. Hu P, Jiang Z, Qu Z, Duan K, Qian D. Serological investigation and prevention and control measures of sheep brucellosis in Pan county. Guizhou Animal Science and Veterinary Medicine 2016; 40(2):27-8 (In Chinese).

64. Niu B, Wei S, Yao P, Wang Y, Gao C, Wang M et al. Serological investigation and prevention and control measures for goat brucellosis in Shizong county. Yunnan Journal of Animal Science and Veterinary Medicine 2007; (6):36 (In Chinese).

65. Li Q, Dong L, Xing H, Li S. Serological detection of goat brucellosis in Shilin County. Journal of Anhui Agricultural Sciences 2013; 41(7):2956-7 (In Chinese).

66. Liu R. Epidemiological investigation of brucellosis in dairy goats in three counties of Yunnan Province and Kappa analysis of serological tests. Master's Thesis*.* Gansu Agricultural University; 2013 (In Chinese).
